# Comparative Evaluation of Dental Implant Failure among Healthy and Well-Controlled Diabetic Patients—A 3-Year Retrospective Study

**DOI:** 10.3390/ijerph17145253

**Published:** 2020-07-21

**Authors:** Mohammed Ghazi Sghaireen, Abdulrahman A. Alduraywish, Kumar Chandan Srivastava, Deepti Shrivastava, Santosh R Patil, Selham Al Habib, May Hamza, Saifulizan Ab Rahman, Edward Lynch, Mohammad Khursheed Alam

**Affiliations:** 1 Prosthodontics, Prosthetic Dental Sciences, College of Dentistry, Jouf University, Sakakah 72345, Saudi Arabia; selham.alruwaili@jodent.org (S.A.H.); dr.may.hamza@jodent.org (M.H.); 2 Department of Internal medicine, Medical College, Jouf University, Sakakah 72345, Saudi Arabia; dr-aaad@ju.edu.sa; 3 Oral Medicine & Radiology, Department of Oral & Maxillofacial Surgery & Diagnostic Sciences, College of Dentistry, Jouf University, Sakakah 72345, Saudi Arabia; drkcs.omr@gmail.com (K.C.S.); dr.santosh.patil@jodent.org (S.R.P.); 4 Periodontics, Department of Preventive Dentistry, College of Dentistry, Jouf University, Sakakah 72345, Saudi Arabia; sdeepti20@gmail.com; 5 School of Dental Sciences, Universiti Sains Malaysia, Kelantan, Kubang Kerian 16150, Malaysia; shaiful@usm.my; 6 Director of Biomedical and Clinical Research, University of Nevada, Las Vegas, NV 89154, USA; edward.lynch@hotmail.com; 7 Orthodontic Division, Preventive Dentistry Department, College of Dentistry, Jouf University, Sakakah 72345, Saudi Arabia; dr.mohammad.alam@jodent.org

**Keywords:** dental implant, diabetes mellitus, dental implant survival, peri-implantitis, implant osseointegration

## Abstract

Diabetes mellitus is known to compromise the various aspects of homeostasis, including the immune response and the composition of oral microflora. One of the oral manifestations of diabetes mellitus is tooth loss and the survival rate of dental implants chosen as a treatment modality for its rehabilitation is controversial. The current study aims to evaluate and compare the failure rate of dental implants between well-controlled diabetic and healthy patients. A retrospective study of case-control design was conceptualized with 121 well-controlled diabetic and 136 healthy individuals. Records of subjects who had undergone oral rehabilitation with dental implants between the periods of January 2013 to January 2016 were retrieved. Post-operative evaluation was carried out for all patients for about three years to assess the immediate and long-term success of the procedure. From a total of 742 dental implants, 377 were placed in well-controlled diabetic patients (case group) and 365 in healthy subjects (control group). A comparable (9.81%), but non-significant (*p* = 0.422) failure rate was found in the case group in comparison to the control group (9.04%). A non-significant (*p* = 0.392) raised number (4.98%) of failure cases were reported among females in comparison to males (4.44%). In respect to arch, the mandibular posterior region was reported as the highest failure cases (3.09%; *p* = 0.411), with 2.29% of cases reported in the mandibular anterior (*p* = 0.430) and maxillary posterior (*p* = 0.983) each. The maxillary anterior region was found to have the least number (1.75%; *p* = 0.999) of failure cases. More (4.98%; *p* = 0.361) cases were reported to fail during the functional loading stage in contrast to osseointegration (4.44%; *p* = 0.365). A well-controlled diabetic status does not impose any additional risk for individuals undergoing dental implant therapy.

## 1. Introduction

Diabetes mellitus (DM) is a pandemic disease with an alarming growth rate [1]. In recently published (2017) statistics on the planetary burden of DM by the International Diabetes Federation (IDF), there are about 425 million adults living with diabetes, and this is estimated to go up to 629 million by 2045 [2]. These growing numbers are translated in terms of raising mortality rate, diminishing quality of life and enormous financial burden incurred on the individual in addition to the government [3]. The number of infants born to conditional diabetic mothers is also on the rise with an estimate of one in five live births in the Middle East and North Africa region (MENA) [2]. With its increasing incidence, gestational diabetes has been classified as a separate entity in addition to the existing type 1 and type 2 DM. In Saudi Arabia, DM is of great concern as it has been ranked seventh on a global platform with about 39 million diabetic people in the country [4]. It is also noteworthy that three (Saudi Arabia, Egypt and United Arab Emirates) out of the top ten countries belong to the MENA region, indicating that DM is not just a national but rather a regional concern.

DM is a complex disorder which flips the metabolism of carbohydrates, proteins and fats from an anabolic to a predominantly catabolic nature. However, hyperglycemia is considered as its primary manifestation due to complete insufficiency in the production or functional disability of insulin [5]. Mismanaged and chronically inflated glycemic levels potentiate to numerous vascular complications affecting small and large blood vessels, which are commonly referred to as microvascular and macrovascular complications [6]. Researchers have identified advanced glycation end-products (AGEs) as the elementary offender for the majority of the complications of DM [7]. The oral cavity can also be compromised under the influence of DM. Among various manifestations, periodontitis has been established as the sixth most common complication of DM [8]. The presence of AGEs in circulation and increased glycemic levels in the periodontal environment leads to immunological malfunctioning, compromised neutrophil activity, and transformation of the oral microflora [9]. The cumulative impact of all said events promotes periodontal breakdown, eventually leading to tooth loss. It is estimated that individuals with diabetes are 1.46 times more likely to have at least one tooth removed compared to those without diabetes [10]. Infrequent dental visits and ignorance towards oral health maintenance contributes further to the problem [11]. The difficulties arising from partial or complete edentulism comprises masticatory insufficiency, occlusal imbalance, temporomandibular joint (TMJ) disorders and further deterioration of the ridge [12].

In the last two decades, dental implant (DI) therapy has emerged as a promising treatment option for restoring the missing teeth. At the same time, there are various local and systemic parameters which can limit the accomplishment of DI therapy. DM is the most widely recognized systemic condition which is viewed as a relative but not absolute contraindication [13]. In the course of assessing the survival rate of DI, effective osseointegration is the first and foremost decisive event following the insertion of the implant. Any compromise in this biological procedure may antagonistically influence immediate or short-term survival, and eventually the overall treatment result [14]. In the subsequent step, where the bone implant integrated unit is subjected to physiological load, it is expected that the adjacent bone will respond and display reactionary bone remodeling in a similar way as before. This phenomenon will appraise long-term survival, which is equally important [15]. The impact of DM on various homeostatic mechanisms, including bone metabolism and the immune system, drives us to assess this condition and its impact on dental implant survival.

The clinical success rate of osseointegrated implants in healthy patients has been studied extensively [16] and the impact of DM on the failure rate of DI is published as well [17]. Although we have some comparative studies assessing implant failures in diabetic and healthy individuals, the results are conflicting [18,19].The current study not only investigated the failure rate (short-term and long-term) of DI, but also analyzed the impact of other factors such as gender and length of implant on failure rate of DI. These multiple parameters make this study stand out from the rest. Hence, the present study is carried out to identify dental implant survival and failure rates in diabetic and healthy patients. The research question, which was intended to be answered by the current study, was “Is there a higher risk of implant failure in well-controlled DM patients when compared with healthy subjects?”

## 2. Materials and Methods

### 2.1. Description of the Study

A hospital-based retrospective study was carried out at the College of Dentistry, the Kingdom of Saudi Arabia (KSA). Ethical approval (04–03–41) was obtained from the local committee of bioethics (LCBE). This retrospective study was conducted according to the principles of the Helsinki Declaration (9th version, 2013).

### 2.2. Sample Description

**Figure 1:**
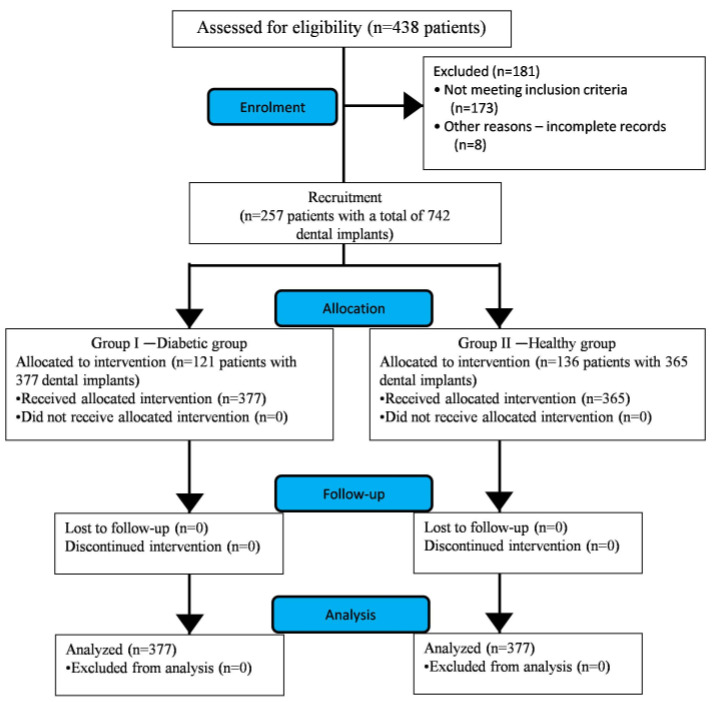
Flow chart showing the study design and recruitment of patients (CONSORT Statement).

Medical history, dental scans and clinical records of patients with partially edentulous arches in the maxilla and/or mandible who later underwent oral rehabilitation with dental implants were retrieved from the period of January 2013 to January 2016. From the implant clinic database, 438 patient profiles were retrieved. Based on the inclusion and exclusion criteria (Table 1), a total of 257 patient profiles with a total of 742 dental implants were found suitable for the study and thus considered for analysis (Figure 1).

The study encompasses of two groups with well-controlled DM patients designated as group I, or “case”, and group II, or “control”, which included healthy subjects. This distinction was based on the evaluation of their glycated hemoglobin (HbA1c) reading at the time of implant placement. As per the American Diabetic Association (ADA), subjects with HbA1c < 6.5% are considered non-diabetic (healthy), whereas patients with HbA1c > 6.5% are labeled as diabetic [20]. Based on the above-mentioned guidelines, participants with HbA1c < 6.5% were enrolled for the control group. Patients with HbA1c levels ranging from 6.5% to 8% were considered as well-controlled diabetic and were enrolled in the case group. Patients with ˃ 8% HbA1c were excluded from the study as they were considered as poorly or uncontrolled diabetics (Table 1). According to the criteria determined for the study groups, 121 and 136 profiles of well-controlled DM patients and healthy subjects were segregated into the control and case group, respectively (Figure 1). The mean age of subjects in the diabetic and healthy groups was 62.41 ± 13.62 years and 59.24 ± 29.36 years, respectively (Table 2).

### 2.3. Study Protocol

Considering the oral findings and dental scans, treatment planning was carried out for the patients in both groups. As per standard protocol, all participants received scaling and root planing (SRP) under phase I of dental therapy. Straumann® Standard Plus (Institut Straumann AG, Basel, Switzerland) dental implants, with sand-blasted and acid-etched implant surfaces (SLA) of different lengths and diameters, were placed according to the manufacturer’s protocols. A trans-gingival healing cap was placed. Subjects in both study groups were prescribed prophylactic antibiotics and post-operative analgesics for a period of 7 days, along with an anti-plaque agent (chlorhexidine 0.12%) for 15 days. After complete healing was observed, a minimum of 16 weeks was given to place the implant-supported fixed dental prosthesis. Patients were instructed to revisit dental clinics every 3 months, especially for soft tissue examination, and every 6 months specifically for the bone response. All patients were followed through the uncovering and final restoration. They were recalled for a regular check-up for 3 consecutive years with an interval of one year after surgery to evaluate the survival of the implants along with the assessment of HbA1c status.

### 2.4. Statistical Analysis

Data were entered into a MS Excel spreadsheet for editing and coding. Characteristics of the sample were described and represented in numbers and percentages. Testing of the hypothesis was performed using the chi square test at a 95% confidence interval (CI). Association was considered statistically significant when *p* < 0.05.

## 3. Results

### 3.1. Descriptive Statistical Analysis Related to Sample

In the present study, a sum of 742 implants were placed in subjects including both study groups. The case group, which consisted of well-controlled diabetic patients, received 377 implants, while the control group got 365. The distribution of implants among the gender cohorts shows females receiving more implants (51.48%) than males (48.51%). On observing the dissipation of implants in accordance with the location, the mandible turned out to be a more popular site (53.77%) than the maxilla (46.22%) (Table 2).

### 3.2. Inferential Statistical Analysis Related to Implant Failure

Bearing the primary aim of the study in mind, implant failure was scrutinized between the study groups. A failure rate of 9.81% was found in the case group, which was higher when compared with the control group (9.04%). However, this result was statistically non-significant (*p* = 0.422) (Table 3).

Firstly, failure rates were observed in the context of gender, where females (4.98%; *p* = 0.392) showed a higher but non-significant failure rate than males (4.44%; *p* = 0.390) (Table 3). Subsequently, inspection of the characteristics of implant failure was done considering the arches. Comparable numbers (maxillary: 4.24% and mandibular: 5.57%) of unsuccessful implants were recorded in the diabetic and control groups (maxillary: 3.83% and mandibular: 5.20%), with the mandible outscoring the maxilla. The variation noted above was not found be to statistically significant (*p* ˃ 0.05) (Table 3).

Another parameter in which we were interested was implant stability. Statistically non-significant but relatively higher numbers of implant failure were reported in the functional loading phase (4.98%; p = 0.361) as compared to osseointegration (4.44%; p = 0.365) (Table 3).

Further exploration of the failure rates was done within the different regions of arches. The posterior region of either jaw had a larger number of failure cases compared to the anterior region (Table 3). Similar observations were made in both groups, which were statistically non-significant (*p* ˃ 0.05) (Table 3).

Lastly, the length of implant was also considered in the series of variables assessed for the comparative evaluation. A comparable number of failure cases in both dimensional categories (<10 mm and >10 mm length) of implant were reported between the groups. A common observation was made in both groups that the majority of non-performing implants were from the category of <10mm implant length. Statistically, all findings with respect to the length of implants were found to be non-significant (<10 mm *p* = 0.918; ˃10 mm *p* = 0.901) (Table 3).

## 4. Discussion

In the present decade, there has been a paradigm shift in patient preference from aesthetics to functional aesthetics. Hence, oral rehabilitation of missing teeth with dental implants has gained popularity.

The literature supports the fact that type 2 DM is more prevalent in the advancing age group with the co-existence of other predisposing factors. As periodontitis is the sixth most common complication of DM, it has become one of the primary reasons for tooth loss in these patients [11,21]. Therefore, the legitimacy of dental implant treatment for these patients must be substantiated.

For the success of rehabilitation of the dental implant, osseointegration is of prime importance. It is the functional healing of the implant surface with the bone tissue without the interposition of connective tissue when they are subjected to functional load. During this process, bone remodeling takes place in the presence of osteoclasts and osteoblasts. It has been reported that during hyperglycemia, there is a change in bone metabolism, as it inhibits osteoblastic differentiation and alters calcium and phosphorous metabolism [22,23]. It has also been shown to change the bone as well as the extracellular matrix [21]. Thus, our concern rises regarding the survival of the implants. In the present study, we have seen that out of 377 dental implants placed in diabetic patients, 17 (4.50%) of them failed after the first stage of surgery, which gave an implant survival rate of 95.49% during the healing period (Table 2). This is in accordance with the observations of Olson et al. [24], Balshi et al. [25], Farzad et al. [26] and, Tawil et al. [27], where the survival was 90%, 94.3%, 96.3% and 97.2% respectively. This could be attributed to the fact that the implant failed during the first stage of healing, meaning that it was not properly osseointegrated. There are various reasons for such changes; one of them is the reduction in bone–implant contact [28]. At the same time, it has also been confirmed that this situation can be reversed if the patient maintained good glycemic control [29]. In our study, we too ensured that the patients included in the case group are well-controlled diabetics. Considering this rationale, patients with HbAc1 levels above 8% at the time of implants were excluded from the study as these patients are considered to be moderately or poorly controlled diabetics. According to Marchand et al., the failure rate of implants in diabetics ranges from 4.4% to 14.3% [30]. In addition to this, patients with other diseases that might affect the result of this study were also excluded. In the control group, 16 out of 365 implants failed during the first stage, with a survival rate of 95.61%. For the current study, while comparing the failure rate of implants between the groups, no significant difference was found. Similar results have also been reported by previous studies [16].

A few studies have quoted that despite higher bone mineral density being seen in femail diabetic patients compared to their male counterparts, females displayed high fracture rates, suggestive of a qualitative difference in the bone. Nonetheless, in our study, the implant failure rate in female (4.98%) patients outscored male counterparts (4.44%) irrespective of the study group, although this was not significant [28,31].

One year after surgery, along with the functional loading, 20 additional implant failures were identified in the diabetic group and 17 in non-diabetic patients, which resulted in an overall, non-significant success rate of 90.18% and 90.95%, respectively. In diabetic patients, Olson et al. [24], Balshi et al. [25] and Farzad et al. [26] noticed an implant success rate of 88.0%, 94.1% and 99.1%, respectively. A success rate of 92.7% was reported even with diabetic geriatric patients [32]. In diabetics, where most failures occurred after the second phase of surgery and during the first year of functional loading, this could be the result of microvascular involvement [26].

In addition to this, in diabetic patients, osteoblastic activity is restricted and parathyroid hormone will alter calcium and phosphorous metabolism. As a result, there will be a decrease in collagen fiber formation and bone cells might suffer apoptosis, which hinders bone formation [33].

In the present study, when intergroup comparisons were done regarding the failure rate in maxillary and mandibular arches, no significant difference was found. Similar results were seen in the study by Alsaadi et al. [34]. When the success rate was analyzed by implant location, the results of our studies were comparable with those of Fiorellini et al. [35]. Although, other studies have found that the survival rate is greater in the mandibular arch when implants are placed anteriorly [33,36].

Considering the similarity between periodontal and peri-implant disease in terms of the etiopathogenesis and clinical outcome, it has been shown that DM might alter the microenvironment around the implant. In the presence of hyperglycemia, immune responses have been shown to be affected and thus a microbiological insult leading to an inflammatory cascade is more likely [37]. This plethora of events will delay the wound healing process and becomes a pathway for soft tissue infection, which will manifest as peri-implant mucositis or peri-implantitis [38,39]. In our study, we found that 4.50% of diabetic patients have peri-implantitis, which is comparable to the previously published studies [17,39]. This implies a higher success rate in our study, which can be substantiated with the fact that the patients in group I were given an anti-plaque agent (chlorhexidine 0.12%). This is similar to a study by Morris et al. [40] where the implant failure rate was reduced from 13.5% to 4.4% when observed for over three years. Regular maintenance has also proved efficient in decreasing the failure rate of implants as a result of this disease. Another factor to consider was antibiotic coverage. These patients were given (in most of cases) a course of prophylactic antibiotics. This result can not conclude that antibiotics alone or synergistically with chlorhexidine has shown these effects, although it is a well-known fact that antibiotics act on bacteria and will thus reduce the bacterial load. Moreover, Michaeli et al. [41] have postulated that prophylactic antibiotics can be a suitable option for the prevention of peri-implantitis. However, in a systematic review by Ting et al. [42], it was summarized that due to the lack of evidence in proper methodologies for various existing studies, a relationship between peri-implantitis and DM cannot be concluded. On the contrary, Monje et al. [43] concluded that patients with diabetes have a higher risk of developing peri-implantitis but not peri-implant mucositis.

When considering the survival rate of implants, osseointegration during the first year and success after functional loading are considered on a long-term basis. Implant survival is basically considered as the end point of treatment. Failed implants were determined as per the Alberktsson criteria [44]. It was seen that in most of cases, implant failure occurs more after functional loading. In our study, we have evaluated implant survival for three years and found that it does not differ significantly when healthy individuals are compared with a diabetic group. In our study, the overall survival rate after three years of follow-up in diabetic and non-diabetic patients was 90.18% and 90.95%, respectively. This was in accordance with other similar studies [15,17,24], although there are also studies where the implant survival rate between healthy and diabetic patients was found to differ significantly [45].

In the present study, the length of the implants had no effect on the survival rate of the implants. These observations were not in accordance with that of Olson et al. [24], who noticed that implant length had a statistically significant relationship with implant survival or failure. On the contrary, in a meta-analysis, Ting et al. concluded that the location and length of the dental implant do not significantly influence its survival [42].

In the present study, considering all the attributes, there was no significant difference in the survival rate of dental implants between well-controlled DM patients and healthy subjects.

There are a few limitations in the present study. Being a retrospective study, the data regarding the duration of DM was not available, which might be a contributing factor in implant failure. Based on the statistical results of the study, the null hypothesis is accepted which states that there is not a higher risk of implant failure between well-controlled DM patients and healthy subjects.

## 5. Conclusions

It can be concluded that well-controlled diabetics do not have any additional risk in the failure rate of dental implants in comparison to healthy individuals, provided they maintain a good glycemic control.

## Figures and Tables

**Table 1:** Inclusion and exclusion criteria for the study.

Inclusion Criteria	Exclusion Criteria
Common Inclusion Criteria: Patients with partially edentulous arches in the maxilla and/or mandible who later underwent oral rehabilitation with dental implants from the period of January 2013 to January 2016	Common Exclusion Criteria: (A) Systemic Subjects <18 years of age Smokers Chronic systemic conditions like hypertension, renal disorders or liver disorders which can influence surgical treatment Chronic medication affecting bone metabolism Osteoporosis (B) Local Intraoral tumor Site of interest having irradiated bone History of bone grafting Cases of immediate loading at the proposed implant site (C) Other Incomplete medical history, dental scans and clinical records of patients
Inclusion Criteria for Diabetic Group I Patient with HbA1c levels ranging from 6.5% to 8% were considered as well-controlled diabetics	Exclusion Criteria for Diabetic Group I Patients having ˃8% HbA1c
Inclusion Criteria for Healthy Group II Subjects with HbA1c < 6.5% are considered non-diabetic	Exclusion Criteria for Healthy Group II Same as common exclusion criteria

**Table 2:** Frequency distribution table showing the descriptive characteristics of the sample.

Variable	Response		Study Group		Total
			Study Group I—Diabetic	Study Group II—Healthy	
Sample Size	No. of patients		121	136	257
	Dental implants placed		377	365	742
Age (expressed as mean ± SD)			62.41 ± 13.62	59.24 ± 29.36	-
Gender		Males	182 (48.27)	178 (48.76)	360 (48.51)
		Females	195 (51.72)	187 (51.23)	382 (51.48)
Location in jaw		Maxilla	175 (46.41)	168 (46.02)	343 (46.22)
		Mandible	202 (53.58)	197 (53.97)	399 (53.77)

SD: Standard Deviation.

**Table 3:** Frequency distribution table showing descriptive and comparative evaluation of implant failure between the study groups.

Variable				Study Groups		Total (742)	*p* Value
				Diabetic (n = 377)	Healthy (n = 365)		
Implant failure rate				37 (9.81)	33 (9.04)	70 (9.43)	0.422
Gender		Males		18 (4.77)	15 (4.10)	33 (4.44)	0.390
		Females		19 (5.03)	18 (4.93)	37 (4.98)	0.392
Implant stability	Osseointegration			17 (4.50)	16 (4.38)	33 (4.44)	0.365
	Functional Loading			20 (5.30)	17 (4.65)	37 (4.98)	0.361
Arch	Maxilla			16 (4.24)	14 (3.83)	30 (4.04)	0.921
	Mandible			21 (5.57)	19 (5.20)	40 (5.39)	0.402
Location in the arch	Maxilla		Anterior	7 (1.85)	6 (1.64)	13 (1.75)	0.999
			Posterior	9 (2.38)	8 (2.19)	17 (2.29)	0.983
	Mandible		Anterior	9 (2.38)	8 (2.19)	17 (2.29)	0.430
			Posterior	12 (3.18)	11 (3.01)	23 (3.09)	0.411
Length of implant	<10 mm			20 (5.30)	17 (4.65)	37 (4.98)	0.918
	>10 mm			17 (4.50)	16 (4.38)	33 (4.44)	0.901
Implant survival rate				340 (90.18)	332 (90.95)	672 (90.56)	0.410

Chi square test (test of significance) is applied at 95% confidence interval (CI).
